# Role of G-Protein-Coupled Receptors in Cardiovascular Diseases

**DOI:** 10.3390/ijms24097760

**Published:** 2023-04-24

**Authors:** Selin Gencer, Emiel P. C. van der Vorst

**Affiliations:** 1 Institute for Cardiovascular Prevention (IPEK), Ludwig-Maximilians-University Munich (LMU), 80336 Munich, Germany; sgencer@altoslabs.com; 2 Altos Labs, Inc., Redwood City, CA 94065, USA; 3 Institute for Molecular Cardiovascular Research (IMCAR), RWTH Aachen University, 52074 Aachen, Germany; 4 Aachen-Maastricht Institute for CardioRenal Disease (AMICARE), RWTH Aachen University, 52074 Aachen, Germany; 5 Interdisciplinary Center for Clinical Research (IZKF), RWTH Aachen University, 52074 Aachen, Germany

Cardiovascular diseases (CVDs), such as ischemic heart disease and stroke, are recognized as major causes of deaths worldwide [1]. The main pathology underlying these diseases, atherosclerosis, is characterized by the formation of lipid laden, inflammatory lesions which can ultimately occlude arteries and block the flow of oxygen-rich blood to vital organs. High cholesterol levels have long been recognized to be a major cause of atherosclerosis development. Therefore, initially, the treatment of this disease has mainly focused on battling hypercholesterolemia [2]. Nevertheless, as also evident by the statistics, lowering lipid levels has not been sufficient so far to curb the increasing rate of CVD-related death. The reason for this may be the notion that dyslipidemia is not the sole factor fueling atherosclerosis; the indispensable role of inflammation in the development of atherosclerotic lesions has been successfully demonstrated by many studies [3]. The inflammatory processes driving atherosclerosis, such as the recruitment of immune cells to sites of inflammation and adhesion of immune cells to the vascular wall, rely heavily on the actions of G-protein-coupled receptors (GPCRs). 

The new Special Issue entitled “Role of G-Protein-Coupled Receptors in Cardiovascular Diseases” of the *International Journal of Molecular Sciences* included a total of five contributions: two original articles and three reviews, providing new information about the roles of GPCRs in CVDs.

The review article by Sundararaman et al. [4] published in this Special Issue beautifully summarized the various roles of the calcium-sensing receptor (CaSR), a GPCR capable of sensing the fluctuations in the levels of extracellular calcium. CaSR is found to be expressed in a variety of tissues with numerous pro-atherosclerotic roles, such as promoting inflammation, vascular calcification, and cardiac fibrosis, among other pathological roles in the cardiovascular system. Such roles of this receptor indicate that its inhibition may be a beneficial target in the treatment of atherosclerotic disease. One of the interesting aspects that is highlighted in this review is the importance of CaSR in the field of hypertension. For example, it is being discussed that CaSR expressed in vascular smooth muscle cells (VSMC) is involved in the regulation of blood pressure via the targeted deletion of CaSR in murine VSMCs under the control of the SM22a promoter [5]. Furthermore, another study highlighted in this review showed a reduction in blood pressure in rats with CaSR activation [6]. These are very important findings because high blood pressure is a recognized risk factor for CVDs [7], and CaSR may thereby be a novel therapeutic target to be further studied to target hypertension and CVD. 

A major regulator of blood pressure is the renin-angiotensin aldosterone system (RAAS), as also discussed by Sutanto et al. in this Special Issue [8]. While a short-term activation of the RAAS, controlling vasoconstriction, is beneficial, its long-term and excessive activation can be detrimental and lead to further cardiovascular complications, such as heart failure. Thus, therapeutic approaches to curb the overactivation of RAAS are being investigated. The review article suggests that combining the inhibition of an enzyme named neprilysin, which degrades the RAAS-correcting natriuretic peptides, together with an angiotensin receptor blocker seems to be an effective treatment of CVDs with an excessive RAAS [8]. Nevertheless, a possible link between this combinatorial treatment approach and cardiac arrythmias could be made; therefore, the search for effective treatments targeting RAAS and blood pressure is still ongoing, and perhaps GPCRs may offer an unexpected, new perspective on this matter. 

Further, on the matter of blood pressure regulation, a study by Maning et al. published in this Special Issue investigated a GPCR adapter protein, βarrestin2, in cardiac contractility [9]. Using in vitro models, a beta blocker medicine, carvedilol, was found to induce contraction in cardiac myocytes through βarrestin2-driven Sarco(endo)plasmic reticulum Ca^2+^-ATPase (SERCA)2a SUMO (small ubiquitin-like modifier)-ylation [9]. However, since this study is purely cell culture based, these findings still require in vivo confirmation in the future.

A detailed review written by Bauer in this Special Issue highlights the roles of the receptor GPR15, a co-receptor for the human immunodeficiency virus (HIV), in blood and vasculature [10]. In this article, it could be shown that a GPR15 ligand named thrombomodulin has anticoagulant characteristics. Thrombosis formation also plays a critical role during atherosclerosis development. In the case of an atherosclerotic lesion rupturing in the vessel wall, platelet aggregation can immediately occlude arteries, or small thrombi can travel through the circulation and lodge themselves in another artery, leading to ischemic disease. Therefore, anticoagulant therapy in clinically significant atherosclerosis is an option to manage thrombosis-related complications. Interestingly, this article also reports findings indicating elevated levels of vascular endothelial cell-derived thrombomodulin fragments in conditions such as cardiovascular and ischemic diseases. As also highlighted in the open questions section of the review article regarding GPR15-related research [10], it is clear that further studies are still needed to fully elucidate the specific roles of GPR15, especially with regards to its effects on atherosclerosis. 

Perhaps a rather-overlooked field in the research of cardiovascular treatment is the impact of the lymphatic system on the development of CVDs among other diseases, such as cancer. As the research article in this Special Issue by Xu and colleagues notes [11], the lymphatic system is found to saturate almost every organ in the body, including the vascular system. Furthermore, among the important roles of the lymphatic system, the regulation of immune cell trafficking is a prominent one, which is an essential process in the development of atherosclerosis. The study by Xu et al. [11] investigated enriched lymphatic GPCRs in human endothelial cells and identified GPRC5B as a significant receptor in the development of lymphatics using in vitro and in vivo models. The study reports that zebrafish and mice lacking this receptor exhibited weakened lymphatic growth. These outcomes are endorsed by the observation that the proliferation and viability of human lymphatic endothelial cells is decreased upon GPRC5B deficiency. Therefore, this study highlights the need for a further understanding of the roles of GPRC5B in the context of disease models.

To summarize, the articles published as part of this Special Issue increase our understanding about the diverse and complex role of GPCRs in CVDs. Although the presented studies provide valuable insights, it is clear that further studies are still required to further elucidate the specific roles of various GPCRs in atherosclerosis and CVDs. 
